# Determining the interviewer effect on CQ Index outcomes: a multilevel approach

**DOI:** 10.1186/1471-2288-10-75

**Published:** 2010-08-19

**Authors:** Sjenny Winters, Mathilde H Strating, Niek S Klazinga, Rudolf B Kool, Robbert Huijsman

**Affiliations:** 1 Kiwa Prismant, P.O. Box 85200, 3508 AE Utrecht, the Netherlands; 2 Erasmus University Rotterdam, institute of Health Policy and Management, Rotterdam, the Netherlands; 3 Department of Social Medicine, Academic Medical Centre, University of Amsterdam, Amsterdam, the Netherlands

## Abstract

**Background:**

The CQ Index for the elderly, a quality-of-care questionnaire administered by conducting interviews, is used to assess clients' experiences in Dutch nursing homes and homes for the elderly. This article describes whether inter-interviewer differences influence the perceived quality of healthcare services reported by residents, the size of this interviewer effect and the influence of the interviewer characteristics on CQ Index dimensions for public reporting.

**Methods:**

Data from 4345 questionnaires was used. Correlations were calculated, reliability analyses were performed, and a multilevel analysis was used to calculate the degree of correlation between two interviewers within one health care institution. Five models were constructed and the Intra Class Correlation (ICC) was calculated. Healthcare institutions were given 1-5 stars on every quality dimensions (1 = worst and 5 = best), adjusted for resident and interviewer characteristics. The effect of these characteristics on the assignment of the stars was investigated.

**Results:**

In a multilevel approach, the ICC showed a significant amount of variance on five quality dimensions. Of the interviewer characteristics, only previous interviewing experience, the reason of interviewing and general knowledge of health care had a significant effect on the quality dimensions. Adjusting for interviewer characteristics did not affect the overall star assignment to the institutions regarding 7 of 12 quality dimensions. For the other five dimensions (Shared decision-making, Meals, Professional competency, Autonomy, and Availability of personnel) a minor effect was found.

**Conclusions:**

We have shown that training, the use of experienced interviewers, written instructions, supervision and educational meetings do not automatically prevent interviewer effects. While the results of this study can be used to improve the quality of services provided by these institutions, several CQ index dimensions should be interpreted with caution for external purposes (accountability and transparency).

## Background

Monitoring the experiences of residents of nursing homes and homes for the elderly is crucial to improve the quality of care and to evaluate the effect of interventions to improve care [1-14]. In an attempt to standardize the method of measuring the experiences of residents in nursing homes and homes for the elderly, in 2006 the Dutch Ministry of Health developed instruments for measuring the experiences of patients in different types of health care facilities [12,15-18]. These questionnaires are based on the CAHPS questionnaires [16]. Also for residents in nursing homes and homes for the elderly, a so called CQ Index, has been developed and pilot-tested [12]. In the Netherlands the nursing homes and homes for the elderly differ: the care given in nursing homes is more intensive than care given in homes for the elderly. Dutch nursing homes and homes for the elderly are obliged to have this survey of residents' opinions conducted every two years. The survey must be administered by an accredited, independent organization. The institutions are ranked for the level of quality and this information is available to the public. The results of the CQ Index serve two purposes. Firstly, it can be used by health care institutions to improve the quality of the services they provide. Secondly, it enlarges the accountability and transparency towards insurers, the Inspectorate for Health Care and future clients.

A commonly used method to assess the healthcare experiences of elderly is a face-to-face interview, in which a standardized questionnaire is administered. Research has shown that face-to-face interviews improve the quality and quantity of the data, and that they are less a burden for respondents when compared to telephone interviews [19,20]. Respondents are more likely to comply with a face-to-face interview than with a telephone interview [21] or a written questionnaire [22]. However, face-to-face interviews do have the possible disadvantage of an interviewer effect, which has been found to be greater than in telephone interviews [23]. There are ways in which interviewers can influence the answers given by respondents to pre-formulated questions [24]. Firstly, interviewers can subconsciously express their own attitudes, opinions, or expectations by means of intonation, verbal and non-verbal communications and non-standard explanation of words as formulated in the interviewer guide [25]. Secondly, elderly respondents are likely to have difficulty choosing one of the pre-defined answer categories. Also, a face-to-face interview is an opportunity for social contact. Therefore, respondents often tend to go into a conversation. As a result, the interviewer has to interpret and translate this into one of the answering possibilities. This interpretation is subjective and may differ between interviewers. This could lead to interviewer bias and false conclusions [22].

While several suggestions have been made to overcome these problems [24,26,27], little is known about how to prevent interviewer effects in face-to-face interviews with elderly [28]. It is known that the quality of data obtained from older individuals may also be affected by the respondent's physical, cognitive, and sensory impairments [29], and it is recognized that face-to-face interviews provide older people with an opportunity for social contact [30]. These studies suggest a special training programme for interviewers before interviewing elderly. Although we do know interviewer effects are likely to influence the results of the survey and several suggestions have been made to diminish this, little is known about which interviewer characteristics cause this effect and how large the effect actually is. In this study, we used the CQ Index to investigate 1) whether experienced interviewers (knowledge of nursing homes and homes for the elderly and more than 70 interviews conducted) influence the perceived quality of healthcare services reported by residents of nursing homes and homes for the elderly in the Netherlands (interviewer effects), 2) the size of the interviewer effect when using interviewers with who conducted a major number of interviews in this study (experienced interviewers) and 3) the influence of the interviewer characteristics on results of the CQ Index dimensions for public reporting. We tried to establish whether structural differences in the scores on the CQ Index between experienced interviewers can be explained by interviewer characteristics, and whether these differences influence how these institutions are ranked for overall quality.

## Methods

Between January 2007 and April 2008 trained interviewers from the accredited research organization, Prismant, administered the CQ Index to residents in 24 nursing homes and 109 homes for the elderly. For this research we asked written permission from all participated these health care institutions to use their CQ Index data for scientific purposes, and all institutions cooperate. This data collection is part of a regular research which is conducted every year in the Netherlands. This method of the research is constructed in a collaboration of relevant stakeholders (Ministry of Health, the branch organization and Inspectorate for Health Care) [12].

### Subjects

The research population consisted of residents of nursing homes and homes for the elderly. Residents who had stayed in the facility for less than 1 month, residents who were very ill, residents with psychiatric conditions, or residents who were convalescing were excluded. In total, 29% of the population met the exclusion criteria. The residents were selected by making a random sample, and tested on representativeness by age and gender.

### Questionnaire: CQ Index

In the first part of the questionnaire, the age, sex, educational level, length of stay and health status of the resident and type of care (nursing home or home for the elderly) was recorded. The central part of the questionnaire consists of 72 questions. Together, these questions represent 15 quality-of-care dimensions (Table 1). All answers were assigned a 1-4 point score, with the higher the score, the less positive the resident experienced the question. The compilation of the scores on the questions to scores on the quality dimensions also resulted in a score ranging from 1 to 4. Means and standard deviations of the scores were calculated. Reliability was measured using Cronbach's alpha (Table 1). The reliability of the dimensions 5, 11, and 13 was low (Cronbach's alpha < 0.6) so these were excluded from further analysis.

**Table 1:** Dimensions of the CQ Index

Dimensions	Number of items	Mean score	sd	Cronbach's α
1. Care plan and evaluation	1	1.71	.939	-
2. Shared decision making	5	2.14	.819	0.81
3. Treatment	4	1.61	.663	0.81
4. Information	6	1.96	.781	0.75
5. Body care	3	1.49	.494	0.55*
6. Meals	1	1.93	.860	-
7. Professional competency	8	1.43	.469	0.82
8. Living comfort	1	1.57	.823	-
9. Atmosphere	4	1.53	.484	0.63
10. Living environment and privacy	4	1.18	.354	0.62
11. Activities	5	1.52	.438	0.54*
12. Autonomy	4	1.52	.647	0.69
13. Mental wellbeing	3	2.19	.531	0.32*
14. Security	1	1.21	.542	-
15. Availability personnel	5	2.16	.581	0.67

* excluded from further analyses

### Interviewers

All interviewers were trained before and during the study - they learned about the content of the questionnaire items and were instructed in interviewing techniques, including the verbal and non-verbal aspects of interviewing. All interviewers received an written interviewer guide, covering the following aspects:

•Preparing for the interview (knowledge of the questionnaire, paying attention to the environment, etc.);

•Introducing and starting the interview (informing the respondent about the duration and the anonymity of their comments);

•The interview itself (how to ask questions, what to do when a respondent does not understand the question or becomes emotional);

•Finishing the interview (informing the respondent about what will be done with the answers).

To minimize interviewer variation, all new interviewers were supervised by experienced interviewers. Meetings were held regularly to allow discussion about the function of interviewing and the robustness of the data collected. At the end of the study, interviewers, Prismant, and institutions discussed about how the interviews had been conducted. In a health care institution 30 interviews were conducted. A resident was interviewed once by one interviewer. In a health care institution a pair of interviewers interviewed all 30 residents. Pairs of interviewers were randomly assigned to the healthcare institutions all over the country with every health care institution a different combination of interviewers. The interviewers who participated in this research have been conducted interviews in at least five health care institutions.

### Interviewer characteristics

Since the research question was whether *experienced *interviewers influence the perceived quality of healthcare services reported by residents, only interviewers were included who conducted at least 70 interviews during this research. The interviewers were asked to complete a questionnaire about a number of characteristics suggested to play a role in interviewer bias [22,23], namely, age, sex, level of education, socioeconomic status, work and previous interviewing experience before this research, general knowledge of healthcare and specific knowledge of care for the elderly in particular (Table 2). Other factors that can possibly influence the outcome of the interview, as determined by an expert panel, were also added to the questionnaire. These were health status, work motivation (intrinsic or economic reasons; an interviewer received € 30,- per completed interview), frequency of interviewing (number of days per month), and whether the interviewers felt uncomfortable with the content of CQ Index.

**Table 2:** Characteristics of the residents (N = 4345)

	%
**Length of stay in a nursing home or home for the elderly**	
< 1/2 year	9.5%
6 months - 1 year	14.0%
1-2 years	18.7%
2-5 year	32.2%
> 5 year	25.5%
**Health status - **good	44.9%
- moderate	45.5%
- poor	9.6%
**Type of care - **homes for the elderly	83,6%
**- **nursing homes	16,4%
**Age **- < 65 years	4.3%
- 65 -74 years	7,5%
- 75 -84 years	34,6%
- > 85 years	53,6%
**Sex **Man	25.3%
Woman	74.7%
**Level of education**	
- no education	3.0%
- lower education	74.6%
- medium education	16.2%
- higher education	6.0%

At the time of data analysis, 4 of the 18 interviewers were no longer traceable and one interviewer had died. The remaining 13 interviewers received the questionnaire, of which 10 were completed and returned. (76.9%).

### Analysis

Inter-interviewer differences in respondents' scores for the quality-of-care dimensions of the CQ Index were assessed using variance analysis. The data we used was cross-classified. The cross-classification was at level 2 (interviewer) with level 1 (residents) and the level 1 units (residents) were also nested in health care institutions (level 2) because the interviewers worked in different health care institutions.

In a multilevel model we investigated the degree of correlation of observations made by interviewers within a health care institution. We also investigated whether the differences in the scores on the dimensions of the CQ Index could be explained by resident characteristics, interviewer characteristics, or by a resident × interviewer interaction. We started with lower level characteristics (resident) before entering higher-level characteristics (interviewer) and the interviewer × resident interaction. Only characteristics that were significantly correlated with the quality dimensions (p ≤ .05) were included in the model. We built a multilevel model in five steps.:

- Model 0: model with no random effects of health care institutions or interviewer

- Model 1: random intercept model (interviewer and institution).

- Model 2: random intercept model, adjusting for resident characteristics.

- Model 3: random intercept model, adjusting for interviewer characteristics.

- Model 4: random intercept model, adjusting for resident characteristics as well as interviewer characteristics.

- Model 5: random intercept model, adjusting for resident and interviewer characteristics and interactions between resident and interviewer.

In all models, all variables were entered as fixed effects.

In Model 5, no interaction effects were found that could be explained by the interaction. Therefore, the interaction effects were excluded from further analysis.

The intra class correlation (ICC) [12,31] was measured as a size of the correlation between observations (interviews with residents) made by interviewers within a institution. The analysis was carried out using SPSS, version 15. Residual analysis was performed and all independent variables were standardized, which enabled comparison of the effects. Deviance tests or likelihood ratio tests were used to compare the relative fit of the different models. The difference in deviance of two nested models has a χ^2 ^distribution with degrees of freedom equal to the number of additional parameters in the larger model. Results were considered statistically significant at a two-sided p ≤ .05 level. The percentage of explained variance was computed.

We gave health care institutions a star on every quality dimension (1 = worst and 5 = best). To assign the stars, we calculated a predicted quality score for each dimension, adjusted for resident characteristics (age, duration of stay, level of education, and health status) [12]. In the next step of the analysis, we corrected the raw scores on all dimensions of the CQ Index for each institution, for the characteristics of the residents (age, duration of stay, educational level, health status) and interviewers (age, educational level, sex and previous interview experience [22]) that were found to be significant. Subsequently, using these scores, all individual institutions were labelled with stars, based on the relative score of an institution in relation to the mean score of all institutions using 95% confidence intervals (CI).

For each institution the number of stars assigned before and after

adjusting for interviewer and resident characteristics were compared and calculated the percentage of institutions that was assigned a different number of stars.

## Results

### Resident and interviewer characteristics

Eighteen interviewers were included. Together they had performed 4345 interviews. On average, an interview lasted 43.2 minutes (sd ± 11.8), and an interviewer carried out 127 interviews; the maximum number of interviews carried out by one person was 512 and the minimum was 70 interviews. The mean age was 83.1 years (sd 11.4), 74.7% was women and 96.4% of the residents was born in the Netherlands. Of the residents 44.9% considered their health to be good, 9.6% as poor, and 45.5% as good neither poor. Other characteristics of the residents are shown in Table 2.

Of the interviewers, two were men. Ninety percent of the interviewers were highly educated, and all were born in the Netherlands. All interviewers had more than 6 years of working experience; 80% more than 10 years. Of 70% of the interviewers, their previous jobs were not related to interviewing (teacher, researcher, engineer, healthcare worker, etc.) (Table 3). In the non-response analysis, there were more men and younger individuals among the non-responders. The mean interview duration was similar between responders and non-responders.

**Table 3:** Characteristics of the experienced interviewers (N = 10)

	%
**Sex **Men	20%
Women	80%
**Age **30-39	10%
40-49	30%
50-59	40%
60-69	20%
**Reason for interviewing **Nice work	30%
Flexible work schedule	20%
Earn money	10%
Useful spending of time	30%
**How many days interviewing per month **2-4 days in a month	40%
5-7 days in a month	20%
8-10 days in a month	20%
> 10 days in a month	10%
**How long interviewing with CQ Index **Between 4 and 6 months	30%
Between 7 and 9 months	20%
More than 9 months	50%
**Previous interview experience **Yes	50%
No	50%
**Knowledge of healthcare **Strongly agree	10%
Agree Disagree	60% 30%
**Knowledge of elderly care **Strongly agree	10%
Agree	70%
Disagree	20%

### Differences in scores on quality dimensions caused by interviewer of resident characteristics

Analysis showed that the scores on the various quality dimensions varied significantly between interviewers (all p < 0.001). All resident characteristics were significantly correlated to at least three dimensions of the CQ Index, whereas previous interviewer experience, sex, reason for interviewing and content of the questionnaire were correlated to two or more dimensions (Table 4).

**Table 4:** Correlations between residents' and interviewers' characteristics on the dimensions of the CQ Index

	Resident characteristics						Interviewer characteristics													
Dimensions	Length of stay	Sex	Age	Education	Health status	Type of care	Sex	Age	Education	Reason	SES	Work experience	Previous Interview experience	How long interviewing	Frequency interviewing	Other jobs	Content questionnaire	Knowledge healthcare	Knowledge elderly care	Health status
1	.01	-.06*	.02	-.05	-.08*	.07*	-.33	.15	-.41	-.20	-.09	.14	.08	.14	.68*	-.23	-.66*	-.06	-.30	-.40
2	-.01	.01	-.01	.03	.11*	.04	-.50	.16	.30	.59*	-.10	-.29	-.18	.21	-.14	.11	.14	.33	.03	-.06
3	.05*	-.02	.02	.04	.15*	.09*	-.06	-.14	.51	.55	.20	-.27	-.61*	.06	-.37	.26	.58*	.14	.07	.23
4	-.14*	-.04	.03	-.03	.01	.10*	-.29	-.39	.64*	-.05	.10	.01	-.53	.24	-.36	.13	.04	.26	.16	.42
6	.06*	-.09*	.03	.05*	.13*	.08*	.61*	-.29	.40	.10	.52	-.13	-.85*	.016	-.17	-.17	.51	-.40	-.28	.62*
7	.05	.01	-.00	.06*	.18*	.04	-.08	-.24	.29	-.26	-.01	-.37	-.54	-.10	-.08	.13	.10	.10	-.03	.10
8	.11*	-.13*	.01	.04	.15*	-.03	.52	-.53	.19	-.19	.03	-.36	-.65*	-.45	-.08	-.01	.07	-.16	.05	.38
9	.00	-.03	-.05*	.07*	.14*	.18*	.33	-.18	.36	.27	.05	-.35	-.68*	-.08	-.31	-.09	.47	.04	.05	.43
10	-.14*	.01	-.05*	.05*	.08*	.33*	-.03	.09	.06	.20	-.22	-.52	.04	-.29	-.20	-.36	-.14	.21	.25	-.18
12	-.03	-.06*	-.06*	.02	.18*	.37*	.56*	.02	.07	.39*	.48	-.39	-.65*	.20	.07	-.12	.35	-.76*	-.62*	.18
14	-.01	-.07*	-.02	-.06*	.10*	.07*	.12	-.81*	.17	-.59*	.12	-.04	-.49	-.57*	-.00	.50	-.22	-.03	.13	-.01
15	.07*	-.04	-.07	.09*	.21*	.11*	.17	-.29	.22	.23	-.03	-.52	-.58*	-.23	-.11	.07	.08	-.09	-.08	.10

1 = Care plan and evaluation 2 = Shared decision-making 3 = Treatment. 4 = Information 6 = Meals 7 = Professional competency 8 = Living comfort 9 = Atmosphere 10 = Living environment and privacy 12 = Autonomy 14 = Security 15 = Availability personnel

Note: Reference category for type of care is homes for the elderly (1).

* is significant at the p ≤ .05 level.

In additional file 1, Table S1, the -2 log likelihood and χ^2 ^of every quality dimension are shown, and decreased from model 1 to model 4. Only characteristics that were significantly correlated to the quality dimensions (p ≤ .05) were included in the model. We determined the -2loglikelihood compared with the previous model.

Table S1, in additional file 1, shows the level of homogeneity between interviewer observations (measured in the same health care institution), explained by interviewer characteristics and resident characteristics on the dimensions. In multilevel analysis, resident characteristics, especially sex, health status and type of care significantly influenced the scores given to the dimensions. Women were more positive than men. Residents with a higher educational level were less positive about several dimensions, as were residents with a better health status. Residents of nursing homes were more negative about healthcare than residents of homes for the elderly. Residents with a higher length of stay were more positive about the information services and the living environment, but were more negative about meals, comfort, and the availability of personnel.

Of the interviewers characteristics, previous interviewing experience was found to significantly affect how residents scored the meals and availability of personnel. The more previous experience the interviewer had, the more negative residents were. On the quality dimension 'autonomy' two interviewer characteristics were found significantly. The more the interviewer did this job for other reasons than economical reasons, the more negative residents were. The more knowledge of health care the interviewers have, the more positive residents were.

Table 5 shows the ICC's of the models. We compared the raw ICC (model 1) with the ICC adjusted for resident and interviewer characteristics (models 2 and 3). The ICC's in model 2 (only resident characteristics) were lower than the raw ICC's for 10 of the twelve quality indicators. Adjustment for resident characteristics is relevant, but the effect on the ICC is minor for the most quality dimensions (max 1.8%). Only for 'Living environment/privacy', the effect is substantial (7.5%).

**Table 5:** ICC on the dimensions of the CQ Index, per model

Dimensions	Model 0 no random intercept	2 Levels	Model 1 random intercept		Model 2 random intercept and level 1 independent variables		Model 3 random intercept and level 2 explanatory variables		Model 4 random intercept with level 1 and 2 variables	
			ICC	Explained variance	ICC	Explained variance	ICC	Explained variance	ICC	Explained variance
1. Care plan and evaluation	0.881	Interv level	0.064	7.39%	0.063	7.30%	0.017	1.97%	0.019	2.18%
		Facility level	0.083	9.54%	0.082	9.48%	0.078	8.94%	0.073	8.42%
2. Shared decision-making	0.671	Interv level	0.119	15.87%	0.113	15.48%	0.120	16.59%	0.120	16.64%
		Facility level	0.060	8.04%	0.060	8.17%	0.036	4.95%	0.036	4.95%
3. Treatment	0.439	Interv level	0.029	8.98%	0.029	8.40%	0.033	10.00%	0.034	10.60%
		Facility level	0.018	5.00%	0.013	3.84%	0.016	4.73%	0.013	3.92%
4. Information	0.61	Interv level	0.073	9.08%	0.072	9.31%	0.027	3.73%	0.029	4.11%
		Facility level	0.059	7.35%	0.052	6.67%	0.095	13.11%	0.089	12.72%
6. Meals	0.74	Interv level	0.026	3.81%	0.026	3.95%	0.006	0.92%	0.004	0.74%
		Facility level	0.055	7.99%	0.054	8.23%	0.055	9.05%	0.059	10.06%
7. Professional competency	0.22	Interv level	0.022	9.09%	0.020	8.62%				
		Facility level	0.015	6.29%	0.014	6.18%				
8. Living comfort	0.677	Interv level	0.028	4.12%	0.024	3.68%	0.023	3.69%	0.019	3.19%
		Facility level	0.068	9.96%	0.066	10.07%	0.073	11.92%	0.073	12.42%
9. Atmosphere	0.234	Interv level	0.024	9.62%	0.023	9.85%	0.024	10.66%	0.022	10.42%
		Facility level	0.025	10.24%	0.018	7.57%	0.024	10.99%	0.020	9.23%
10. Living environment/privacy	0.125	Interv level	0.002	19.51%	0.002	23.61%				
		Facility level	0.028	32.24%	0.014	19.51%				
12. Autonomy	0.418	Interv level	0.036	8.12%	0.034	9.64%	0.000	0.00%	0.002	0.54%
		Facility level	0.116	26.39%	0.049	13.82%	0.092	23.52%	0.051	15.32%
14. Security	0.294	Interv level	0.008	26.23%	0.009	3.20%	0.000	0.07%	0.001	0.49%
		Facility level	0.004	14.50%	0.002	5.89%	0.001	0.40%	0.000	0.10%
15. Availability personnel	0.338	Interv level	0.028	6.58%	0.029	7.29%	0.020	5.41%	0.025	6.73%
		Facility level	0.046	10.98%	0.035413	8.89%	0.018	11.70%	0.038	10.45%

ICC **= Intra Class Correlation, recorded as % of explained variance by variables included in the model**

The ICC's of model 3 (interviewer characteristics) were lower then the ICC's of model 1 for five of the ten quality dimensions. Adjusting for interviewer characteristics also shows limited decrease of the ICC's (with max 4.7%). On five of the ten quality dimensions the ICC's were increasing, but not substantial (max 1.3%).

The ICC's of model 4 were lower than the raw ICC's in model 1 in five of the ten quality dimensions (max 4.9).

### Differences in star assignment to institutions

We calculated to what extent interviewer characteristics (as part of the interviewer effect) affected the overall star assignment to the healthcare institutions (table 6). Interviewer and resident characteristics did not affect the star assignment for any institutions for seven of the CQ Index dimensions, changed the star assignment to 1 of the 133 institutions (0.8%) of the three CQ Index dimensions "Meals", "Autonomy", and "Availability personnel" and altered the star assignment to 3 of the 133 institutions (2.3%) of the CQ Index dimension "Shared decision-making", and altered the star assignment to 13,5% of the institutions of the CQ Index dimension "Professional competency".

**Table 6:** Changes in star assignments to institutions for the care of the elderly

Dimensions	Changes in scores	Total percentage of discrepancy	Total nursing homes	Total homes for the elderly
Care plan and evaluation	0	0.00%	0	0
Shared decision-making	3 of 133 institutes	2.26%	0	3
Treatment	0	0.00%	0	0
Information	0	0.00%	0	0
Meals	1 of 133 institutes	0.75%	1	0
Professional competency	10 of 74 institutes	13.51%	0	10
Living comfort	0	0.00%	0	0
Atmosphere	0	0.00%	0	0
Living environment/privacy	0	0.00%	0	0
Autonomy	1 of 133 institutes	0.75%	0	1
Security	0	0.00%	0	0
Availability personnel	1 of 133 institutes	0.75%	0	1

## Discussion

We investigated whether characteristics of interviewers who conducted a major number of interviews influenced the way the residents of nursing homes and homes for the elderly scored the dimensions of the CQ Index, which measures residents' experience of the healthcare services provided. Despite their experience, the use of a standard questionnaire, training, supervision and educational meetings, we still detected significant interviewer effects. We investigated whether this effect could be explained by the characteristics of the interviewers, characteristics of the residents, or by an interaction between residents and interviewers. However, interviewer sex, age, education, socioeconomic status, work experience, how long and the frequency of interviewing, other jobs, health status and knowledge of elderly care did not explain this variation, and thus these characteristics are not a major source of interviewer bias. Only previous interviewing experience, the reason of interviewing and general knowledge of health care had a limited influence on the scores given to the different CQ Index dimensions. Possibly, certain dimensions are open to more interpretation than others. The differences we found, despite the fact they are experienced interviewers, may possibly be related to other characteristics, such as skills, presentation, and intonation during the interview [22]. Future research should evaluate these variables, for example by using observational techniques.

We also investigated the impact of the interviewer characteristics (as part of the interviewer effect) on public reporting. Interviewer characteristics did not substantially influence public reporting through star assignment based on the CQ Index dimensions, with exception of the quality dimensions 'Professional competency' and 'Shared-decision making'. Further research should more extensively determine the impact of the interviewer effect on star assignment to the health care institutions.

The interviewer effect can be reduced in several ways. Firstly, the questions in the questionnaire regarding the CQ Index dimensions that had high ICC's should be reformulated to prevent interpretation differences. Secondly, special attention should be paid to instructing interviewers by the research organizations that conduct the CQ Index surveys, especially on the dimensions with high ICC's and the dimensions that we found to influence the star assignment. To diminish the risk of interviewer effects on the quality dimension 'Professional competency', the 30 interviews could be conducted by three, interviewers. This, however, requires more organizational efforts and will lead to higher costs.

A limitation of this research was the poor reliability of several quality dimensions and the lack of variability in other quality dimensions. The pattern of findings could be a result of the multiple comparisons we made. Another limitation was the small number of interviewers (n = 10) who reported the characteristics themselves. Furthermore, they all worked for the same organization (Prismant). A small number of interviewers could lead to large error effects [32]. The experience of the interviewers filtered beginners' mistakes, which also can lead to interviewer effects. Further research should duplicate our study including more interviewers and more residents, including interviewers from different research organizations (introducing another level of possible interviewer effects) and interviewers with less experience. Ranking institutions with a multilevel approach with several levels: resident, interviewer, research organization and health care institution (cross level classified design) can determine the impact of the interviewer effects on the CQ Index dimensions for public reporting and can give suggestions for a minimum of conducted interviews.

## Conclusions

We have shown that training, the use of experienced interviewers, interview guides, supervision and educational meetings do not automatically prevent interviewer effects. Data control during and after the investigation is still necessary. Our findings suggest that the results for some CQ Index dimensions ("Professional competency" and "Shared-decision making") published on a public website should be interpreted with caution, especially when used for accountability and transparency. This can be done by combining the CQ Index results with additional information from other sources (for example healthcare indicators) to provide a more complete and balanced view of the quality of healthcare organizations. Other quality dimensions are reliable enough for accountability and transparency despite the influence of the interviewer.

## Competing interests

The authors declar that they have no competing interests.

## Authors' contributions

SW performed the design of the research, collected data and performed the multilevel analysis. MS contributed in the analysis and the interpretation of data. TK, NK and RH have been involved in writing, reading and commenting on the manuscript and assisted in the design of the study. All authors read and approved the final manuscript.

## Pre-publication history

The pre-publication history for this paper can be accessed here:

http://www.biomedcentral.com/1471-2288/10/75/prepub

## Supplementary Material

Additional file 1 **Table S1 Study results of the interviewer effect on the dimensions of the CQ Index, per model**. Click here for file
