# Supplementary material for: Determining the interviewer effect on CQ Index outcomes: a multilevel approach
Source: BMC Med Res Methodol. 2010 Aug 19;10:75. doi: 10.1186/1471-2288-10-75 (PMC2936930; doi:10.1186/1471-2288-10-75)
Supplement: Additional file 1 — Table S1 Study results of the interviewer effect on the dimensions of the CQ Index, per model. [file 1471-2288-10-75-S1.DOC]

## Additional file 1, Table S1 Study results of the interviewer effect on the dimensions of the CQ Index, per model

| Dimension 1  Care plan and evaluation | Model 1 | | | Model 2 | | Model 3 | | | model 4 | |
| --- | --- | --- | --- | --- | --- | --- | --- | --- | --- | --- |
|  | random intercept | |  | random intercept and level 1 independent variables |  | random intercept and level 2 explanatory variables | |  | random intercept with level 1 and 2 variables |  |
| Fixed effects | *Coeff* | *SE* | | *Coeff* | *SE* | *Coeff* | *SE* | | *Coeff* | *SE* |
| Level 1 (resident) |  |  | |  |  |  |  | |  |  |
| Intercept | 1.654* | 0.055 | | 1.801* | 0.072 | 1.527* | 0.214 | | 1.709* | 0.231 |
| Sex |  |  | | -0.120* | 0.037 |  |  | | -0.134* | 0.051 |
| Health status |  |  | | -0.042* | 0.016 |  |  | | -0.081* | 0.023 |
| Type of care |  |  | | 0.062* | 0.028 |  |  | | 0.095* | 0.033 |
| Level 2 (interviewer) |  |  | |  |  |  |  | |  |  |
| Content questionnaire |  |  | |  |  | -0.159 | 0.074 | | -0.170 | 0.078 |
| Frequency interviewing |  |  | |  |  | 0.102 | 0.053 | | 0.099 | 0.055 |
| -2Log likelihood / χ²(df) | 7858.276 |  | | 7578.990* | 93.095 (3) | 4367.169* | 3211.822(1) | | 4221.785* | 48.461(3) |

*** is significant at the p ≤ .05 level**

Note: Reference category for type of care is homes for the elderly (1).

| Dimension 2  Shared decision-making | Model 1 | | | Model 2 | | | Model 3 | | | | model 4 | |
| --- | --- | --- | --- | --- | --- | --- | --- | --- | --- | --- | --- | --- |
|  | random intercept | |  | random intercept and level 1 independent variables |  | | random intercept and level 2 explanatory variables | | |  | random intercept with level 1 and 2 variables |  |
| Fixed effects | *Coeff* | *SE* | | *Coeff* | | *SE* | *Coeff* | | *SE* | | *Coeff* | *SE* |
| Level 1 (resident) |  |  | |  | |  |  | |  | |  |  |
| Intercept | 2.186* | 0.067 | | 2.168* | | 0.066 | 1.623* | | 0.365 | | 1.630* | 0.365 |
| Health status |  |  | | 0.116* | | 0.014 |  |  | | | 0.080* | 0.021 |
| Level 2 (interviewer) |  |  | |  | |  |  |  | | |  |  |
| Reason |  |  | |  | |  | 0.485 | 0.239 | | | 0.476 | 0.238 |
| -2Log likelihood /χ² (df) | 7245.821 |  | | 7150.241* | | 95.580(1) | 3381.454* | 3768.786 (1) | | | 3349.911* | 31.544(1) |

*** is significant at the p ≤ .05 level**

| Dimension 3  Treatment | Model 1 | | | Model 2 | | Model 3 | | | model 4 | |
| --- | --- | --- | --- | --- | --- | --- | --- | --- | --- | --- |
|  | random intercept | |  | random intercept and level 1 independent variables |  | random intercept and level 2 explanatory variables | |  | random intercept with level 1 and 2 variables |  |
| Fixed effects | *Coeff* | *SE* | | *Coeff* | *SE* | *Coeff* | *SE* | | *Coeff* | *SE* |
| Level 1 (resident) |  |  | |  |  |  |  | |  |  |
| Intercept | 1.576* | 0.034 | | 1.568* | 0.034 | 1.501* | 0.108 | | 1.504* | 0.110 |
| Duration of stay |  |  | | 0.041* | 0.009 |  |  | | 0.038 | 0.012 |
| Health status |  |  | | 0.091* | 0.009 |  |  | | 0.074 | 0.012 |
| Type of care |  |  | | 0.072* | 0.013 |  |  | | 0.051 | 0.016 |
| Level 2 (interviewer) |  |  | |  |  |  |  | |  |  |
| Content questionnaire |  |  | |  |  | 0.105 | 0.070 | | 0.098 | 0.070 |
| Former interview experience |  |  | |  |  | 0.085 | 0.090 | | 0.079 | 0.092 |
| -2Log likelihood /χ²(df) | 7079.455 |  | | 6647.509* | 143.982(3) | 3784.538* | 2862.971(1) | | 3610.602* | 57.979(3) |

*** is significant at the p ≤ .05 level**

| Dimension 4  Information | Model 1 | | Model 2 | | Model 3 | | | model 4 | |
| --- | --- | --- | --- | --- | --- | --- | --- | --- | --- |
|  | random intercept |  | random intercept and level 1 independent variables |  | random intercept and level 2 explanatory variables | |  | random intercept with level 1 and 2 variables |  |
| Fixed effects | *Coeff* | *SE* | *Coeff* | *SE* | *Coeff* | *SE* | | *Coeff* | *SE* |
| Level 1 (resident) |  |  |  |  |  |  | |  |  |
| Intercept | 2.142* | 0.056 | 2.135* | 0.056 | 2.056* | 0.065 | | 2.055* | 0.066 |
| Duration of stay |  |  | -0.078* | 0.016 |  |  | | -0.089* | 0.020 |
| Type of care |  |  | 0.071* | 0.024 |  |  | | 0.063 | 0.034 |
| Level 2 (interviewer) |  |  |  |  |  |  | |  |  |
| Level of education |  |  |  |  | 0.138 | 0.063 | | 0.132 | 0.065 |
| -2Log likelihood /χ²(df) | 7628.700 |  | 7321.162* | 153.769(2) | 4128.469* | 3192.693(1) | | 3982.392* | 73.038(2) |

*** is significant at the p ≤ .05 level**

| Dimension 6  Meals | Model 1 | | | Model 2 | | Model 3 | | | model 4 | |
| --- | --- | --- | --- | --- | --- | --- | --- | --- | --- | --- |
|  | random intercept |  | | random intercept and level 1 independent variables |  | random intercept and level 2 explanatory variables | |  | random intercept with level 1 and 2 variables |  |
| Fixed effects | *Coeff* | | *SE* | *Coeff* | *SE* | *Coeff* | *SE* | | *Coeff* | *SE* |
| Level 1 (resident) |  | |  |  |  |  |  | |  |  |
| Intercept | 1.962* | | 0.038 | 2.127* | 0.054 | 1.879* | 0.126 | | 2.117* | 0.130 |
| Duration of stay |  | |  | 0.080* | 0.013 |  |  | | 0.054* | 0.017 |
| Sex |  | |  | -0.133* | 0.031 |  |  | | -0.161* | 0.039 |
| Education |  | |  | 0.027* | 0.014 |  |  | | 0.031 | 0.018 |
| Health status |  | |  | 0.114* | 0.013 |  |  | | 0.092* | 0.017 |
| Type of care |  | |  | 0.034 | 0.023 |  |  | | 0.040 | 0.027 |
| Level 2 (interviewer) |  | |  |  |  |  |  | |  |  |
| Sex |  | |  |  |  | 0.025 | 0.049 | | 0.035 | 0.046 |
| Former interview experience |  | |  |  |  | 0.147* | 0.042 | | 0.151* | 0.040 |
| Health status |  | |  |  |  | 0.006 | 0.083 | | -0.022 | 0.079 |
| -2Log likelihood /χ² (df) | 10332.070 | |  | 9555.378* | 155.338(5) | 5689.529* | 1932.925(2) | | 5313.526* | 75.201(5) |

*** is significant at the p ≤ .05 level**

| Dimension 7  Professional competency | Model 1 | | | Model 2 | | Model 3 | | model 4 | |
| --- | --- | --- | --- | --- | --- | --- | --- | --- | --- |
|  | random intercept | |  | random intercept and level 1 independent variables |  | random intercept and level 2 explanatory variables |  | random intercept with level 1 and 2 variables |  |
| Fixed effects | *Coeff* | *SE* | | *Coeff* | *SE* | *Coeff* | *SE* | *Coeff* | *SE* |
| Level 1 (resident) |  |  | |  |  |  |  |  |  |
| Intercept | 1.489* | 0.031 | | 1.471* | 0.029 |  |  |  |  |
| Education |  |  | | 0.020* | 0.010 |  |  |  |  |
| Health status |  |  | | 0.098* | 0.009 |  |  |  |  |
| Level 2 (interviewer) |  |  | |  |  |  |  |  |  |
| -2Log likelihood /χ² (df) | 3367.186 |  | | 3161.547* | 102.819(2) |  |  |  |  |

*** is significant at the p ≤ .05 level**

| Dimension 8  Living comfort | Model 1 | | | Model 2 | | Model 3 | | | model 4 | |
| --- | --- | --- | --- | --- | --- | --- | --- | --- | --- | --- |
|  | random intercept |  | | random intercept and level 1 independent variables |  | random intercept and level 2 explanatory variables | |  | random intercept with level 1 and 2 variables |  |
| Fixed effects | *Coeff* | | *SE* | *Coeff* | *SE* | *Coeff* | *SE* | | *Coeff* | *SE* |
| Level 1 (resident) |  | |  |  |  |  |  | |  |  |
| Intercept | 1.616* | | 0.040 | 1.877* | 0.051 | 1.550 | 0.056 | | 1.817* | 0.068 |
| Duration of stay |  | |  | 0.070* | 0.012 |  |  | | 0.071* | 0.015 |
| Sex |  | |  | -0.215* | 0.028 |  |  | | -0.209* | 0.034 |
| Health status |  | |  | 0.118* | 0.012 |  |  | | 0.118* | 0.015 |
| | Level 2 (interviewer) |  |  |  |  |  |  |  |  | | --- | --- | --- | --- | --- | --- | --- | --- | --- | |  | |  |  |  |  |  | |  |  |
| Former interview experience |  | |  |  |  | 0.095 | 0.052 | | 0.096 | 0.048 |
| -2Log likelihood /χ² (df) | 10013.042 | |  | 9693.556* | 106.495 (3) | 5419.472* | 2137.042 (2) | | 5222.062* | 65.803(3) |

*** is significant at the p ≤ .05 level**

| Dimension 9  Atmosphere | Model 1 | | | Model 2 | | Model 3 | | model 4 | |
| --- | --- | --- | --- | --- | --- | --- | --- | --- | --- |
|  | random intercept | |  | random intercept and level 1 independent variables |  | random intercept and level 2 explanatory variables |  | random intercept with level 1 and 2 variables |  |
| Fixed effects | *Coeff* | *SE* | | *Coeff* | *SE* | *Coeff* | *SE* | *Coeff* | *SE* |
| Level 1 (resident) |  |  | |  |  |  |  |  |  |
| Intercept | 1.560* | 0.032 | | 1.559* | 0.031 | 1.533* | 0.052 | 1.529* | 0.050 |
| Age |  |  | | -0.005 | 0.008 |  |  | -0.004 | 0.010 |
| Education |  |  | | 0.010 | 0.008 |  |  | 0.015* | 0.011 |
| Health status |  |  | | 0.070* | 0.008 |  |  | 0.053* | 0.010 |
| Type of care |  |  | | 0.078* | 0.013 |  |  | 0.066* | 0.016 |
| Level 2 (interviewer) |  |  | |  |  |  |  |  |  |
| Former interview experience |  |  | |  |  | 0.119 | 0.051 | 0.114 | 0.049 |
| -2Log likelihood /χ² (df) | 4617.334 |  | | 4282.336* | 83.749(4) | 2370.275* | 637.354(3) | 2223.267* | 36.752(4) |

*** is significant at the p ≤ .05 level**

| Dimension 10  Living environment/privacy | Model 1 | | Model 2 | | Model 3 | | model 4 | |
| --- | --- | --- | --- | --- | --- | --- | --- | --- |
|  | random intercept |  | random intercept and level 1 independent variables |  | random intercept and level 2 explanatory variables |  | random intercept with level 1 and 2 variables |  |
| Fixed effects | *Coeff* | *SE* | *Coeff* | *SE* | *Coeff* | *SE* | *Coeff* | *SE* |
| Level 1 (resident) |  |  |  |  |  |  |  |  |
| Intercept | 1.127* | 0.016 | 1.123* | 0.013 |  |  |  |  |
| Duration of stay |  |  | -0.017* | 0.004 |  |  |  |  |
| Age |  |  | 0.008 | 0.004 |  |  |  |  |
| Education |  |  | 0.002 | 0.004 |  |  |  |  |
| Health status |  |  | 0.019* | 0.004 |  |  |  |  |
| Type of care |  |  | 0.108* | 0.010 |  |  |  |  |
| -2Log likelihood /χ²(df) | 395.023 |  | 241.624* | 38.350(4) |  |  |  |  |

*** is significant at the p ≤ .05 level**

| Dimension 12  Autonomy | Model 1 | | | Model 2 | | Model 3 | | | model 4 | | |
| --- | --- | --- | --- | --- | --- | --- | --- | --- | --- | --- | --- |
|  | random intercept |  | | random intercept and level 1 independent variables |  | random intercept and level 2 explanatory variables | |  | random intercept with level 1 and 2 variables |  | |
| Fixed effects | *Coeff* | | *SE* | *Coeff* | *SE* | *Coeff* | *SE* | | *Coeff* | | *SE* |
| Level 1 (resident) |  | |  |  |  |  |  | |  | |  |
| Intercept | 1.512* | | 0.045 | 1.664* | 0.046 | 1.402* | 0.111 | | 1.714* | | 0.135 |
| Sex |  | |  | -0.134* | 0.020 |  |  | | -0.134* | | 0.029 |
| Age |  | |  | 0.025* | 0.009 |  |  | | 0.027* | | 0.013 |
| Health status |  | |  | 0.105* | 0.009 |  |  | | 0.106* | | 0.013 |
| Type of care |  | |  | 0.256* | 0.020 |  |  | | 0.207* | | 0.025 |
| Level 2 (interviewer) |  | |  |  |  |  |  | |  | |  |
| Sex |  | |  |  |  | -0.085 | 0.048 | | -0.111 | | 0.056 |
| Reason |  | |  |  |  | 0.178* | 0.052 | | 0.108 | | 0.063 |
| Former interview experience |  | |  |  |  | 0.072 | 0.042 | | 0.038 | | 0.047 |
| Knowledge of healthcare |  | |  |  |  | -0.373* | 0.084 | | -0.358 | | 0.100 |
| Knowledge of elderly |  | |  |  |  | 0.095 | 0.055 | | 0.024* | | 0.067 |
| -2Log likelihood /χ² (df) | 7064.407 | |  | 6494.537* | 142.468(4) | 3321.278* | 3173.258(1) | | 3071.494* | | 49.957(5) |

*** is significant at the p ≤ .05 level**

| Dimension 14  Security | Model 1 | | Model 2 | | Model 3 | | | model 4 | |
| --- | --- | --- | --- | --- | --- | --- | --- | --- | --- |
|  | random intercept |  | random intercept and level 1 independent variables |  | random intercept and level 2 explanatory variables | |  | random intercept with level 1 and 2 variables |  |
| Fixed effects | *Coeff* | *SE* | *Coeff* | *SE* | *Coeff* | *SE* | | *Coeff* | *SE* |
| Level 1 (resident) |  |  |  |  |  |  | |  |  |
| Intercept | 1.229* | 0.020 | 1.358* | 0.031 | 1.183* | 0.069 | | 1.248* | 0.094 |
| Sex |  |  | -0.105* | 0.019 |  |  | | -0.068* | 0.027 |
| Education |  |  | -0.010 | 0.009 |  |  | | -0.034* | 0.012 |
| Health status |  |  | 0.044* | 0.008 |  |  | | 0.040* | 0.011 |
| Type of care |  |  | 0.062* | 0.009 |  |  | | 0.043* | 0.012 |
| Level 2 (interviewer) |  |  |  |  |  |  | |  |  |
| Age |  |  |  |  | -0.037 | 0.023 | | -0.041 | 0.030 |
| Reason |  |  |  |  | -0.001 | 0.041 | | 0.006 | 0.052 |
| How long interviewing |  |  |  |  | -0.005 | 0.016 | | -0.004 | 0.019 |
| -2Log likelihood /χ²(df) | 6842.411 |  | 6245.011* | 149.350(4) | 2863.587* | 3381.424(1) | | 2577.569* | 71.504(4) |

*** is significant at the p ≤ .05 level**

| Dimension 15  Availability personnel | Model 1 | | Model 2 | | Model 3 | | | model 4 | | |
| --- | --- | --- | --- | --- | --- | --- | --- | --- | --- | --- |
|  | random intercept |  | random intercept and level 1 independent variables |  | random intercept and level 2 explanatory variables | |  | random intercept with level 1 and 2 variables |  | |
| Fixed effects | *Coeff* | *SE* | *Coeff* | *SE* | *Coeff* | *SE* | | *Coeff* | | *SE* |
| Level 1 (resident) |  |  |  |  |  |  | |  | |  |
| Intercept | 1.966* | 0.037 | 1.951* | 0.036 | 1.922* | 0.051 | | 1.911* | | 0.055 |
| Duration of stay |  |  | 0.063* | 0.010 |  |  | | 0.060* | | 0.013 |
| Education |  |  | 0.024* | 0.010 |  |  | | 0.027* | | 0.014 |
| Health status |  |  | 0.124* | 0.010 |  |  | | 0.107* | | 0.013 |
| Type of care |  |  | 0.094* | 0.018 |  |  | | 0.064* | | 0.021 |
| Level 2 (interviewer) |  |  |  |  |  |  | |  | |  |
| Work experience |  |  |  |  | 0.121 | 0.049 | | 0.115 | | 0.053 |
| Former interview experience |  |  |  |  | -0.102* | 0.047 | | -0.095 | | 0.051 |
| -2Log likelihood /χ²(df) | 6973.528 |  | 6390.533* | 145.749(4) | 3757.638* | 1316.448(2) | | 3485.782* | | 67.964(4) |

**is significant at the p ≤ .05 level**
